# Anti-TRPV2 Autoantibody Linked to Sudden Infant Death Syndrome

**DOI:** 10.1161/CIRCULATIONAHA.125.073748

**Published:** 2025-07-08

**Authors:** Ange Maguy, Agnès Tessier, Yuvaraj Mahendran, Manon Denis, Benjamin Lauzier, Flavien Charpentier, Jin Li

**Affiliations:** Department of Physiology, University of Bern, Switzerland (A.M., J.L.).; Nantes Université, CHU Nantes, CNRS, INSERM, L’institut du Thorax, France (A.T., M.D., B.L., F.C.).; PEPperPRINT GmbH, Heidelberg, Germany (Y.M.).

**Keywords:** autoantibodies, autoimmunity, infant mortality, sudden infant death syndrome

As a leading cause of infant death, sudden infant death syndrome (SIDS) remains a perplexing diagnosis with no clear underlying biological substrate.^1^ In the past decade, studies have emerged demonstrating that circulating autoantibodies targeting cardiac antigens can underlie life-threatening arrhythmias.^2^ Because autoimmunity as a cause of SIDS has not yet been explored, we screened infant serum samples for the presence of autoantibodies targeting cardiac ion channels and examined how immunoglobulins may play a driving role in the pathogenesis of SIDS.

Comparing cases of SIDS and accidental suffocation and strangulation in bed with healthy controls, we established the autoantibody profile of 47 serum samples using peptide microarray (Figure [A]), as previously described.^2^ Strikingly, only 1 single autoantibody targeting the transient receptor potential vanilloid 2 (TRPV2) channel (PTGPNATESVQPMEGQEDEG) was significantly associated with SIDS (*P*=0.028 versus controls, the default correction in limma). Collectively, we detected anti-TRPV2 autoantibodies in 84.6% of infants with SIDS compared with 50.0% in cases of accidental suffocation and strangulation in bed and 25.0% in controls.

**Figure.**
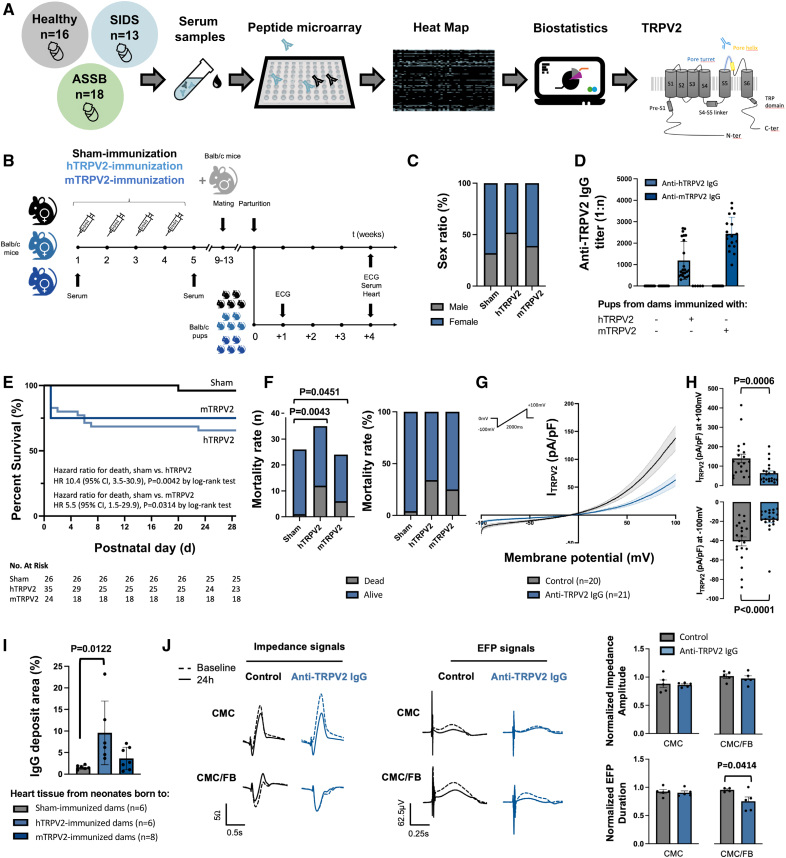
**Immunosignature of SIDS. A**, Serum samples were collected from infants with sudden infant death syndrome (SIDS; age at death, 4.2±3.0 months), infants with accidental suffocation and strangulation in bed (ASSB; age at death, 3.9±4.0 months), and healthy infants and analyzed for autoantibodies against cardiac ion channel antigens with peptide microarray (PEPperPRINT GmbH). SIDS is defined as the sudden unexpected death of an infant <1 year of age that remains unexplained after a thorough case investigation, including a complete autopsy along with a neuropathological report (confirming the absence of structural brain abnormalities), postmortem toxicology and metabolic analysis, review of the circumstances of death, and clinical history according to the consensus from the 3rd International Congress on Sudden Infant and Child Death.^1^ The ASSB group consisted of infants with accidental suffocation/strangulation (eg, positional asphyxia, overlay) as the presumed cause of death, as determined by the local assigned medical examiner. Genetic screening was performed in only 2 ASSB cases, and test results were negative. The general heat map of immunoglobulin G (IgG) responses against a set of 100 extracellular epitope sequences between groups is shown. Data were background corrected and variance-stabilizing normalized (VSN) for 1-way ANOVA. False discovery rate was controlled at 10% cutoff with the Benjamini-Hochberg method. VSN data were then further explored with the limma approach. Using univariate logistic regression model, we found that the presence of anti-transient receptor potential vanilloid 2 (TRPV2) autoantibodies was linked to SIDS with an accuracy estimated at 71% (odds ratio, 1.71 [95% CI, 1.15–2.53]; β coefficient, 0.536; SE, 0.200; *P*=0.007). Anti-TRPV2 autoantibodies target the channel pore turret. **B**, Animal study design. Female Balb/c mice (Charles River Laboratories France) were immunized with human (hTRPV2) or murine (mTRPV2) TRPV2 peptide or KLH (sham). After mating with male Balb/c mice, they gave birth to pups. **C**, Sex ratio of pups born to immunized mice. **D**, Anti-TRPV2 antibody titer on postnatal day 28. **E**, Kaplan-Meier survival curve. Log-rank (Mantel-Cox) test was performed to analyze the statistical significance and to estimate the hazard ratio for death. **F**, Postnatal mortality rate. Categorical variables were compared with the Fisher exact test. **G**, TRPV2 currents were recorded on HEK293 cells stably expressing hTRPV2 channels with a Nanion Port-a-Patch mini planar patch-clamp system. Represented are mean current densities in response to a voltage ramp under control condition (mean cell capacitance, 23.2±4.3pF) and with anti-TRPV2 IgG (mean, 35.4±5.3 pF). SEM values are plotted as shaded areas. Cell capacitance did not differ between groups (*P*=0.09, Mann-Whitney *U* test). **H**, Mean±SEM current densities recorded at −100 and +100 mV in hTRPV2-HEK293 cells under control condition vs anti-TRPV2 IgG. *P* values were calculated with the Mann-Whitney *U* test. **I**, Mean myocardial IgG infiltration in neonates born to immunized dams. Data were compared by 1-way ANOVA followed by the Dunnett post hoc test. **J**, Representative traces of impedance and extracellular field potential (EFP) signals recorded in human induced pluripotent stem cell–derived cardiomyocytes (CMCs; iCell Cardiomyocytes^2^ FUJIFILM Cellular Dynamics, Inc) and fibroblasts (FBs; iCell Cardiac Fibroblasts FUJIFILM Cellular Dynamics, Inc) with the Nanion CardioExcyte 96 system. Cells were treated with anti-TRPV2 IgG vs vehicle, at baseline (dashed lines) vs 24 hours (solid lines). Bar graphs show normalized impedance amplitude and EFP duration data averaged over 5 wells per condition ±SEM. For impedance data, a Kruskal-Wallis test was run followed by a Dunn multiple-comparisons test. EFP data were compared by 1-way ANOVA followed by the Tukey post hoc test.

TRPV2 channel is a homotetramer with a cation-permeable pore allowing a Ca^2^^+^ influx into cells.^3^ A long extracellular loop (pore turret) is characteristic of TRPV2 and represents an exclusive binding site for extracellular ligands (Figure [A]).^3^ The target epitope of the autoantibody we identified precisely matches the pore turret, a region that essentially contributes to the stabilization of the open conformation of the channel.^3^ In the heart, TRPV2 channels prevail in cardiomyocytes, macrophages, and fibroblasts.^4^

To determine whether a causal relationship exists between TRPV2 autoantibodies and SIDS, female Balb/c mice were immunized against the human TRPV2 channel target sequence or the murine equivalent (Figure [B]). All female mice were then mated with male mice. After an uneventful gestation period, pups were born in equal numbers and comparable sex ratio (Figure [C]). The detection of anti-TRPV2 immunoglobulin G in pups provided evidence for the transplacental passage of maternal anti-TRPV2 autoantibodies to neonates (Figure [D]). Twelve of 35 and 6 of 24 live-born neonates from dams with human TRPV2 and murine TRPV2 autoantibodies, respectively, died during the first postnatal month, whereas 1 offspring died in the sham group (*P*=0.0174; Figure [E and F]). Most deaths occurred early, within 6 postnatal days. Kaplan-Meier survival analysis computed a 10-fold increased risk of postnatal death with human TRPV2 autoantibodies (*P*=0.0042) and 5-fold increased mortality in the presence of murine TRPV2 autoantibodies (*P*=0.0314). Previous studies have shown that TRPV2 plays a critical role in the integrity of intercalated disks, which is vital for coordinating the transmission of mechanical forces between neighboring cardiomyocytes.^4^ However, no structural heart disease was evident in our neonatal mice with TRPV2 autoantibodies. Similar observations were made in functional TRPV2-knockout mice lacking exclusively the pore region of the channel.^4,5^

To better understand the mechanisms underlying sudden death in TRPV2-seropositive pups, TRPV2-mediated currents were recorded with the patch-clamp technique. Heterologous expression of human TRPV2 channels in HEK293 cells under mechanical stimulation resulted in an inward current at −100mV, which reversed to become a large outward rectifying current at voltages positive to −10mV. In the presence of TRPV2 autoantibodies, TRPV2 whole-cell currents were reduced by half (*P*=0.0006; Figure [G and H]). To elucidate the role of autoantibody-mediated TRPV2 blockade in cardiac cells, we used a hybrid system that allows an impedance readout (correlating with cell contractility) in combination with extracellular field potential recordings (correlating with the QT interval on ECG). Because immunostaining revealed anti-TRPV2 deposition within the myocardium of pups (Figure [I]), in a pattern that coincides with spindle-shaped fibroblasts interspersed between cardiomyocytes, human induced pluripotent stem cell–derived cardiomyocytes were cocultured with human induced pluripotent stem cell–derived cardiac fibroblasts. TRPV2 antibodies significantly shortened extracellular field potential duration when human induced pluripotent stem cell–derived cardiomyocytes and fibroblasts were cocultured (Figure [J]), thus portending a crucial role of heterocellular interactions in TRPV2-mediated arrhythmogenesis.

According to the triple-risk model, SIDS occurs in a biologically vulnerable infant during a critical developmental period intersected by environmental stressors.^1^ Several pathomechanisms have been proposed ranging from primary electrical diseases to serotonin-related brainstem dysfunction, metabolic conditions, epilepsy, and inflammation.^1^ In line with the multifactorial perspective of SIDS, the presence of TRPV2 autoantibodies would compound other intrinsic/extrinsic factors to cause sudden death. Given the impact of SIDS on families, large prospective cohort studies are needed to validate TRPV2 autoantibody as the first biomarker able to identify infants at risk. Moreover, it may be valuable in unclear sudden death cases to help discern a presumably mechanical (accidental suffocation) from a biological (autoimmunity) cause. In addition, circulating TRPV2 autoantibodies, as new causative pathogens of SIDS, also present a potential target for novel treatment approaches, for example, through autoantibody removal/neutralization.

Study data and methods will be available on reasonable request to the corresponding author. Institutional review board approvals were obtained from each recruiting site, and only infants whose parents gave written informed consent were enrolled. The animal study was approved by institutional animal care and use committees.

## Article Information

### Acknowledgments

The authors are immensely grateful to Prof Thomas G. Blanchard and the National Institute of Health NeuroBioBank for providing serum samples of patients with sudden unexpected infant death. They are grateful for the relentless support provided by the team of PEPperPRINT GmbH, in particular Sarah Schott. They also extend thanks to the Sophistolab AG team (especially Anja Schöpflin) for the outstanding histological staining and to the BIOTEM (in particular Georgia Alaimo and Sandra Caillet) and innoVitro GmbH team (in particular Peter Linder, Dr Matthias Goßmann, Dr Bettina Lickiss) for the excellent collaboration. The authors acknowledge Nantes University Health Research Institute animal facility and Therassay core facility (SFR François Bonamy, Nantes, France), member of the Scientific Interest Group (GIS) Biogenouest, and IBISA.

### Sources of Funding

This work was supported by a Spark (CRSK-3_190182 to Dr Li), Ambizione (PZ00P3_173961 to Dr Li), and Eccellenza (PCEFP3_203333 to Dr Li) grant from the Swiss National Science Foundation.

### Disclosures

Dr Li reports previous employment by BioMarin Pharmaceutical Inc, outside the submitted work. Dr Maguy reports previous consultant fees from BioMarin Pharmaceutical Inc, outside the submitted work. The other authors report no conflicts.

## References

[1] Goldstein RD Blair PS Sens MA Shapiro-Mendoza CK Krous HF Rognum TO Moon RY; 3rd International Congress on Sudden Infant and Child Death. Inconsistent classification of unexplained sudden deaths in infants and children hinders surveillance, prevention and research: recommendations from the 3rd International Congress on Sudden Infant and Child Death. Forensic Sci Med Pathol. 2019;15:622–628. doi: 10.1007/s12024-019-00156-9 31502215 10.1007/s12024-019-00156-9 PMC6872710

[2] Maguy A Tardif J-C Busseuil D Ribi C Li J. Autoantibody signature in cardiac arrest. Circulation. 2020;141:1764–1774. doi: 10.1161/CIRCULATIONAHA.119.044408 32312099 10.1161/CIRCULATIONAHA.119.044408

[3] Dosey TL Wang Z Fan G Zhang Z Serysheva II Chiu W Wensel TG. Structures of TRPV2 in distinct conformations provide insight into role of the pore turret. Nat Struct Mol Biol. 2019;26:40–49. doi: 10.1038/s41594-018-0168-8 30598551 10.1038/s41594-018-0168-8 PMC6458597

[4] Entin-Meer M Keren G. Potential roles in cardiac physiology and pathology of the cation channel TRPV2 expressed in cardiac cells and cardiac macrophages: a mini-review. Am J Physiol Heart Circ Physiol. 2020;318:H181–H188. doi: 10.1152/ajpheart.00491.2019 31809212 10.1152/ajpheart.00491.2019

[5] Park U Vastani N Guan Y Raja SN Koltzenburg M Caterina MJ. TRP vanilloid 2 knock-out mice are susceptible to perinatal lethality but display normal thermal and mechanical nociception. J Neurosci. 2011;31:11425–11436. doi: 10.1523/JNEUROSCI.1384-09.2011 21832173 10.1523/JNEUROSCI.1384-09.2011 PMC3192449

